# Role of Nurse Practitioners in Caring for Patients With Complex Health Needs

**DOI:** 10.1097/MLR.0000000000001364

**Published:** 2020-09-10

**Authors:** Taressa K. Fraze, Adam D.M. Briggs, Elizabeth K. Whitcomb, Kristen A. Peck, Ellen Meara

**Affiliations:** * Dartmouth Institute for Health Policy and Clinical Practice, Geisel School of Medicine, Dartmouth College, Lebanon, NH; † Department of Family and Community Medicine; ‡ Philip R. Lee Institute for Health Policy Studies, University of California San Francisco, San Francisco, CA; § Warwick Medical School, Division of Health Sciences, University of Warwick, Coventry; ∥ The Health Foundation, 8 Salisbury Square, London, UK; ¶ Dartmouth Hitchcock Health, Lebanon, NH; # National Bureau of Economic Research, Cambridge, MA; ** Department of Health Policy and Management, Harvard T. H. Chan School of Public Health, Harvard University, Boston, MA

**Keywords:** workforce, nurse practitioners, primary care, complex patients, team-based care

## Abstract

**Objective:**

The objective of this study was to estimate trends in the percentage of Medicare beneficiaries cared for by nurse practitioners from 2012 to 2017, to characterize beneficiaries cared for by nurse practitioners in 2017, and to examine how the percentage of beneficiaries cared for by nurse practitioners varies by practice characteristics.

**Design:**

An observational study of 2012–2017 Medicare fee-for-service beneficiaries’ ambulatory visits. We computed the percentage of beneficiaries with 1 or more ambulatory visits from nurse practitioners and the percentage of beneficiaries receiving the plurality of their ambulatory visits from a nurse practitioner versus a physician (ie, predominant provider). We compared beneficiary demographics, clinical characteristics, and utilization by the predominant provider. We then characterized the predominant provider by practice characteristics.

**Key Results:**

In 2017, 28.9% of beneficiaries received any care from a nurse practitioner and 8.0% utilized nurse practitioners as their predominant provider—an increase from 4.4% in 2012. Among beneficiaries cared for by nurse practitioners in 2017, 25.9% had 3 or more chronic conditions compared with 20.8% of those cared for by physicians. Beneficiaries cared for in practices owned by health systems were more likely to have a nurse practitioner as their predominant provider compared with those attending practices that were independently owned (9.3% vs. 7.0%).

**Conclusions:**

Nurse practitioners are caring for Medicare beneficiaries with complex needs at rates that match or exceed their physician colleagues. The growing role of nurse practitioners, especially in health care systems, warrants attention as organizations embark on payment and delivery reform.

## Abstract 2

Supplemental Digital Content is available in the text.

Providers, payers, and policymakers^1^,^2^ increasingly focus on caring for the growing number of patients with complex health needs^3^–^6^—including those with multiple chronic conditions or other serious illness. These patients have disproportionately high health care costs^7^,^8^ and may require more intensive and frequent care which places a greater burden on primary care providers.^4^,^9^–^11^ New payment and delivery models,^12^–^16^ like the Comprehensive Primary Care Plus program,^13^ target primary care to ensure patients, especially those with complex health needs, have coordinated care that is high quality and cost-effective.

To meet increased needs, practices have turned to nurse practitioners to extend capacity.^17^–^19^ Evidence supports the notion that nurse practitioners provide care that is comparable to physicians in terms of quality,^20^–^22^ utilization,^21^,^23^ and satisfaction^21^ metrics. The debate remains, however, on the role nurse practitioners should have in caring for patients with clinically complex health needs.^20^,^24^,^25^ In one study, 28% of physicians reported that in their practice, nurse practitioners offer care for complex patients, conversely, 68% of nurse practitioners say they care for complex patients.^26^,^27^ At the same time, around half (40%–65%) of nurse practitioners report having their own patient panels.^28^–^30^ Patient assignment to providers versus nurse practitioners may be driven by physician preferences, organizational leadership, and relationships between nurse practitioners and physicians rather than patient complexity or competence of the nurse practitioner.^31^–^33^ Some argue that nurse practitioners should care for patients with routine clinical needs because these patients may benefit from protocol-based care,^34^ which would, in turn, allow physicians to spend more time with patients who are clinically complex^35^–^37^ and might require the deep knowledge physicians acquire through a decade of medical training.^36^,^38^ Conversely, others argue that nurse practitioners may be most effective at providing in-depth, frequent care that focuses on regular management and coordination of patients’ complex clinical needs, which draws upon communication and coordination skills.^34^,^39^

The debate surrounding the appropriate role for nurse practitioners in primary care lacks evidence about which patients currently receive care from nurse practitioners^40^ and whether nurse practitioner roles vary with system or practice characteristics. To address these knowledge gaps, we first estimated trends from 2012 to 2017 in the percentage of Medicare beneficiaries for whom nurse practitioners deliver the plurality of primary care (ie, for whom nurse practitioners are the predominant provider) to show how roles are changing over time. Then, for 2017, we describe beneficiary characteristics (including clinical complexity) comparing beneficiaries according to their predominant provider of primary care, nurse practitioner versus physician. Finally, we examine whether the percentage of beneficiaries cared for by nurse practitioners differs according to organizational characteristics and geographic settings.

## METHODS

### Data Sources and Study Population

We used 2012–2017 Medicare physician and outpatient claims for beneficiaries aged 18 and older, residing in a US state or Washington, DC, and continuously enrolled in fee-for-service Parts A and B. We excluded beneficiaries who turned 65 in a given year to ensure continuous enrollment.

We used a novel linkage of Medicare fee-for-service claims with OneKey database produced by the health care analytic firm, IQVIA to compare beneficiaries cared for by their predominant provider. OneKey is a commercial database which characterizes medical practices in terms of ownership relationships, number of physicians, and provider composition (specialty and professional training). OneKey is a proprietary database that utilizes the American Medical Association’s Physician Masterfile, publicly available sources, and primary data collection efforts.

### Assigning Beneficiaries to Providers and Practices

Similar to the methodology used by the Centers for Medicare and Medicaid Services (CMS) in value-based programs, such as the Medicare Shared Savings Program, we used a set of eligible office or ambulatory evaluation and management codes to assign beneficiaries to providers and practices.^41^,^42^ Evaluation and management codes are used by both primary care and specialist providers when delivering routine, evaluation services to new or established patients. These codes, regardless of the provider specialty, are considered “primary care” services by CMS.^41^ Specific eligible codes as well as outpatient settings are detailed in Appendix (Table 1, Supplemental Digital Content 1, http://links.lww.com/MLR/C71).

We assigned beneficiaries to the clinician delivering the plurality of their ambulatory visits. We excluded beneficiaries who received the plurality of their care from a clinical nurse specialist or a physician assistant since these providers may deliver care to different patient populations and in different settings than nurse practitioners.

We then assigned beneficiaries to the practice site (single site location) where they received the plurality of their qualifying ambulatory visits. Practices were defined using IQVIA’s OneKey data linked with claims to group providers (using national provider identifiers and tax identification numbers) into practices. Practice exclusions are described in the Appendix (page 2, Supplemental Digital Content 1, http://links.lww.com/MLR/C71).

Similar to CMS’ approach, beneficiaries were first assigned to the primary care provider (defined as family practice, general practitioner, internal medicine, and geriatrician physicians, nurse practitioners, certified nurse specialists, and physician assistants) from whom they received the plurality of their visits. Then any beneficiaries without any visits to a primary care provider were assigned to the specialist physician who delivered the plurality of their visits.

### Measures

**FIGURE 1:**
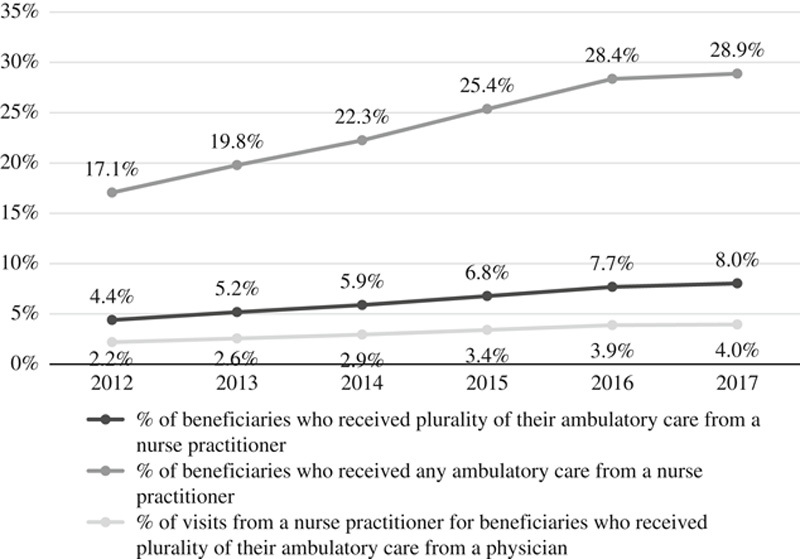
Percentage of ambulatory care provided by nurse practitioners to Medicare beneficiaries from 2012 to 2017.

We first computed measures to characterize the role of nurse practitioners delivering ambulatory care services for Medicare beneficiaries from 2012 to 2017. We used 3 annual measures of beneficiary care received from nurse practitioners: (1) the percentage of beneficiaries with 1 or more qualifying visit from a nurse practitioner; (2) the percentage of beneficiaries who received the plurality of their qualifying visits from a nurse practitioner; and (3) the percentage of qualifying visits nurse practitioners provided to beneficiaries who received the plurality of their care from a physician (Fig. 1).

**FIGURE 2:**
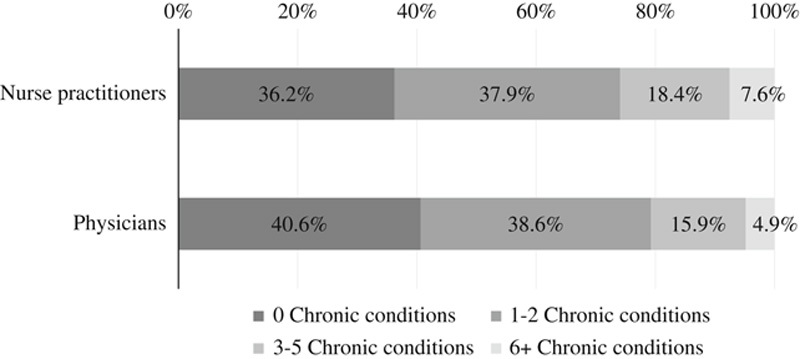
Percentage of beneficiaries receiving the plurality of their ambulatory care from nurse practitioners compared with physicians, by complexity, in 2017.

Using 2017 claims, we then compared the demographic, geographic, and clinical characteristics for beneficiaries according to their predominant provider (nurse practitioner or physician) shown in Table 1 and Figure 2. Beneficiary demographic characteristics, created from claims, included mean age, sex, race, disabled status, and if the beneficiary was dual-eligible for Medicare and Medicaid. Area-level characteristics, including median household income, percentage of residents under the poverty level, and urbanicity, were created using beneficiary ZIP Code and US Census Bureau’s American Community Survey. Finally, clinical characteristics were derived using hierarchical condition categories (HCCs) which are used to adjust payments to Medicare Advantage plans for expected spending.^43^ We used HCCs to group beneficiaries by clinical complexity (0 HCCs, 1–2 HCCs, 3–5 HCCs, 6+ HCCs).

**TABLE 1:** Characteristics of Beneficiaries Who Get the Plurality of Their Care From Nurse Practitioners Versus Physicians, 2017

Characteristics	Total (N=23,502,189)	Nurse Practitioners (n=2,026,111)	Physicians (n=21,476,078)
Demographic characteristics			
Mean age (y)	71.7	70.4	71.9
Over 85 (%)	10.5	13.7	10.1
Under 65 (%)	14.0	21.5	13.2
Female (%)	56.2	61.5	55.6
White, non-Hispanic (%)	82.7	84.1	82.4
Black, non-Hispanic (%)	8.5	9.5	8.5
Other, non-Hispanic (%)	3.8	2.4	4.0
Hispanic ethnicity (%)	5.0	4.0	5.1
Disabled (original reason for Medicare eligibility) (%)	22.0	31.3	21.0
Dual-eligible for Medicaid (%)	18.8	31.7	17.6
Died in calendar year (%)	4.2	8.4	3.8
Area characteristics			
Median household income ($)	57,913	52,085	58,559
Residents under poverty level (%)	12.9	14.4	12.7
Isolated rural (%)	4.5	7.3	4.1
Small town (%)	6.9	10.1	6.5
Micropolitan (%)	13.0	18.0	12.4
Metropolitan (%)	75.7	64.6	77.1
Clinical characteristics (%)			
Mean number of hierarchical condition categories	1.5	1.9	1.5
Congestive heart failure (%)	9.8	12.7	9.7
Coronary artery disease (%)	4.8	5.0	4.9
Diabetes (%)	24.9	25.1	24.1
Cancer (%)	9.4	6.4	10.0
Chronic obstructive pulmonary disease (%)	10.0	12.9	9.9
End-stage renal disease (%)	1.2	1.3	1.2

We used 2017 data to compute, separately according to beneficiaries’ predominant provider of ambulatory care, several measures of service utilization: inpatient stays, inpatient observation stays, emergency department visits and inpatient stays for ambulatory care sensitive conditions (defined using the Agency for Healthcare and Quality’s prevention quality indicators^44^). Finally, we described Medicare payments for acute care stays, other inpatient combined long-term care and skilled nursing facility, and other (including durable medical equipment, imaging, evaluation, and management visits, procedures, tests, outpatient facilities, home health agency, hospice, and unclassified). Measure definitions are shown in the Appendix (Table 2, Supplemental Digital Content 1, http://links.lww.com/MLR/C71).

**FIGURE 3:**
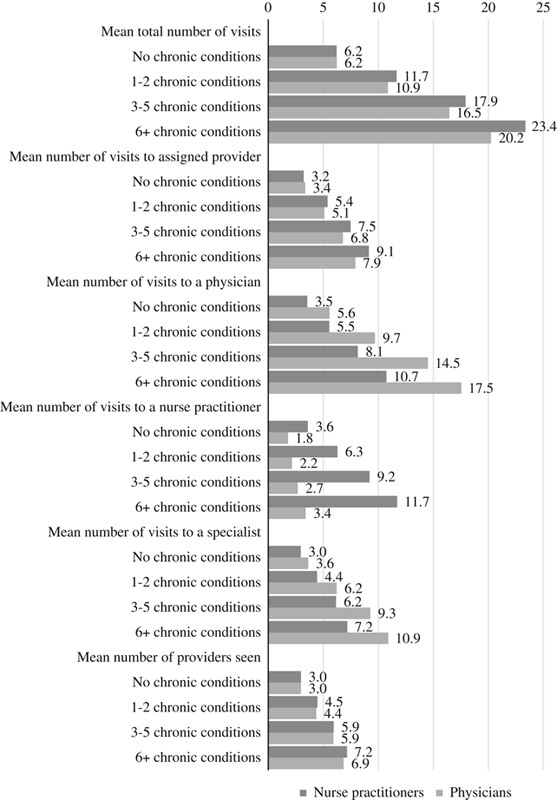
Ambulatory care visits for beneficiaries who get the plurality of their care from nurse practitioners versus physicians, by complexity, in 2017.

As shown in Figure 3, we used 2017 data to compare beneficiaries’ visits to their assigned provider type versus other providers according to their predominant provider of care (nurse practitioners vs. physicians), stratifying beneficiary groups by clinical complexity (0 HCCs, 1–2 HCCs, 3–5 HCCs, 6+ HCCs).

**FIGURE 4:**
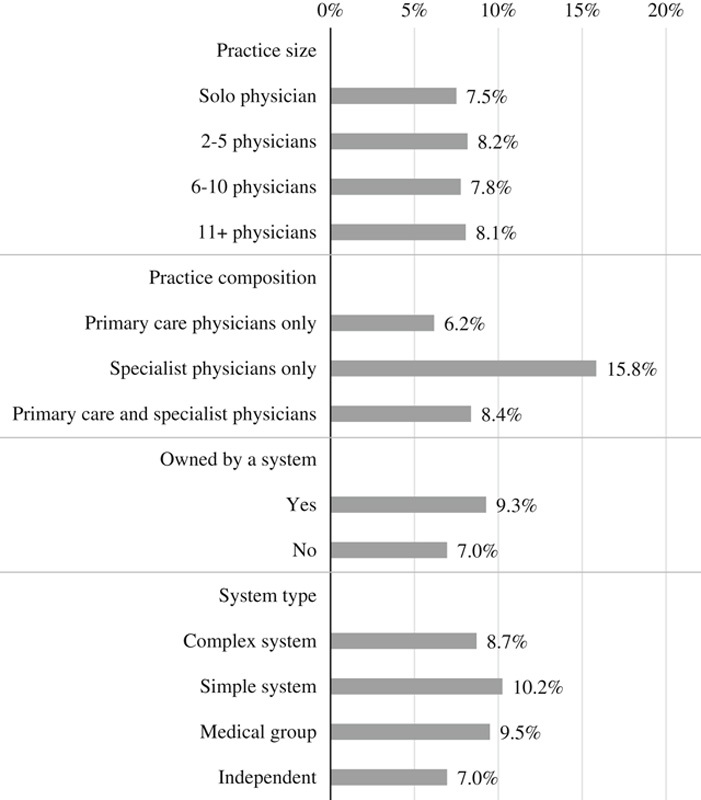
Percentage of beneficiaries who get the plurality of their care from nurse practitioners by practice characteristics in 2017.

Using 2017 data from OneKey, for beneficiaries with nurse practitioners versus physicians as a predominant provider, we compared the following practice-level characteristics: (1) number of physicians; (2) whether the practice included specialist physicians; (3) whether the practice was part of a system (owned by a corporate parent); and (4) the type of system that owned the practice (Fig. 4). Type of system that owned the practice was defined as: (1) complex integrated systems that included hospitals, medical groups, and owner subsidiaries; (2) simple integrated systems that included hospitals and medical groups; (3) medical group systems that did not include hospitals; and (4) independently owned (ie, the practice was not owned by a system).

### Statistical Analysis

We calculated descriptive statistics to: (1) determine the percentage of beneficiaries with nurse practitioners as their predominant provider of outpatient evaluation visits from 2012 to 2017; (2) compare the demographic and clinical characteristics of beneficiaries assigned to nurse practitioners versus physicians in 2017; (3) identify how the percentage of beneficiaries with nurse practitioners as their predominant provider of outpatient evaluation visits varies by practice-level characteristics in 2017; and (4) identify variation across states in the percentage of beneficiaries with nurse practitioners as their predominant provider.

For our main beneficiary outcomes of interest, we estimated adjusted means by known correlates as described below. We estimated the percent of beneficiaries receiving a plurality of care from nurse practitioners versus physicians by a number of chronic conditions, adjusted for demographic characteristics, and area-level poverty, income, and urbanicity using negative binomial regression (Fig. 2). We estimated the volume of ambulatory visits by the number of chronic conditions adjusted for beneficiary demographics, area-level poverty, income, and urbanicity, and HRR using linear regressions (obtained by interacting provider assignment with a categorical variable indicating the number of HCCs; Fig. 3). Finally, we estimated measures of service utilization in inpatient and outpatient settings adjusted for beneficiary demographic and clinical characteristics, area-level poverty, income, and urbanicity, and HRR using linear regressions (Appendix, Tables 4–12, Supplemental Digital Content 1, http://links.lww.com/MLR/C71). Beneficiary demographic, area-level poverty, income, urbanicity, and clinical characteristics included in the models are shown in Table 1. Since our research questions were descriptive in nature, we used models to adjust dependent variables for known correlates rather than to identify the strongest predictors of dependent variables. Independent variables were entered into models simultaneously. Full regression results are shown in the Appendix (Tables 3, 5–12, 14–19, 21, Supplemental Digital Content 1, http://links.lww.com/MLR/C71).

This study was approved by Dartmouth’s Committee for the Protection of Human Subjects.

## RESULTS

### Growth in Use of Nurse Practitioners

The proportion of Medicare beneficiaries who received care from nurse practitioners increased steadily from 2012 to 2017. In 2017, 28.9% of Medicare beneficiaries had at least 1 visit with a nurse practitioner, up from 17.1% in 2012. Similarly, the proportion of beneficiaries who received the plurality of their care from nurse practitioners increased from 4.4% in 2012 to 8.0% in 2017 (Fig. 1). Among beneficiaries with a nurse practitioner as their predominant provider, the proportion of visits with a nurse practitioner remained stable from 2012 to 2017 (59.6%–62.2%); among beneficiaries with a physician as a predominant provider, the proportion of visits conducted by a nurse practitioner was modest but increasing (increased from 2.2% in 2012 to 4.0% in 2017; Fig. 1).

### Characteristics of Beneficiaries

Medicare beneficiaries who get the plurality of their care from nurse practitioners tended to be more clinically and socially complex. Nurse practitioners cared for greater proportions of beneficiaries who were: under 65 (primarily disabled adults), over 85, original reason for entitlement for Medicare was disability, dually eligible for Medicaid, died during the year, and residents of areas with lower median income, higher rates of poverty, and in rural locations. Beneficiaries cared for by nurse practitioners were also more clinically complex with a higher mean number of HCCs (Table 1).

### Beneficiaries With Complex Clinical Needs

After adjusting for demographics and HRR of residence, beneficiaries with a nurse practitioner as their predominant provider were more likely to have 3 or more chronic conditions than beneficiaries with a physician as their predominant provider (25.9% vs. 20.8%, respectively; Fig. 2, Appendix, Table 3, Supplemental Digital Content 1, http://links.lww.com/MLR/C71).

### Hospital Utilization

After adjusting for beneficiary characteristics, beneficiaries who received care from a nurse practitioner versus a physician had similar hospital-based utilization; the mean number of emergency department visits was 0.44 versus 0.44, and inpatient stays was 0.29 versus 0.29, and potentially avoidable inpatient stays was 0.52 versus 0.52. Mean total payments for beneficiaries who received care from a nurse practitioner was $10,644 compared with $10,145 who received care from a physician—most of the difference on total payments was driven by long-term care and skilled nursing facility payments ($1667 vs. $970). Full results are shown in the Appendix (Tables 4–12, Supplemental Digital Content 1, http://links.lww.com/MLR/C71).

### Ambulatory Care Visits

Beneficiaries’ patterns of ambulatory visits varied based on their predominant provider and clinical complexity. Adjusting for beneficiary demographics and HRRs, clinically complex beneficiaries who received the plurality of their care from nurse practitioners had more ambulatory visits than beneficiaries who receive the plurality of their care from physicians (Fig. 3). For example, beneficiaries with 6 or more HCCs who had a nurse practitioner as a predominant provider received 23.4 visits on average, compared with 20.2 visits for similar beneficiaries with a physician as a predominant provider. As the number of chronic conditions increased, the percentage of ambulatory visits delivered by the predominant provider (nurse practitioner or physician) decreased. Full results are shown in the Appendix (Tables 13–19, Supplemental Digital Content 1, http://links.lww.com/MLR/C71).

### Practice Characteristics

The percentage of beneficiaries who received the plurality of their care from nurse practitioners varied by practice characteristics. Practices with specialist physicians and nonsolo physician practices had greater percentages of beneficiaries receiving the plurality of their care from nurse practitioners. Practices owned by a system had higher percentages of beneficiaries receiving the plurality of their care from nurse practitioners compared with practices not in a system (9.3% vs. 7.0%; Fig. 4).

### State Variations

The percentage of beneficiaries with a nurse practitioner as their predominant provider varied by US state. States in the northwest and northeast had the highest percentages of beneficiaries with nurse practitioners as their predominant provider. States with the lowest percentages were geographically dispersed and included California, Hawaii, Florida, and others (map and state percentages shown in Appendix, Table 20, Supplemental Digital Content 1, http://links.lww.com/MLR/C71).

## DISCUSSION

By 2017, over 1 in 4 Medicare beneficiaries received some ambulatory care from a nurse practitioner—a nearly 70% rise from 2012. For a small, but growing, group of beneficiaries, 8.0% in 2017, nurse practitioners were the predominant providers of care. As a group, beneficiaries relying on nurse practitioners were more clinically and, as in prior research, more socially^26^,^27^ complex than those receiving care from physicians. These differences remained after controlling for differences in the region of beneficiary residence. Finally, nurse practitioners were more likely to deliver care in larger, multispecialty practices.

Our findings on the increasing role of nurse practitioners are supported by prior research, with one study finding that the number of nurse practitioners in physician practices increased to nearly a quarter of providers in 2016.^18^ Similarly, steady growth is projected in the workforce of nurse practitioners with an estimated annual growth of 6.8% from 2016 to 2030 compared with just 1.1% estimated growth for physicians^17^ in the same period. Given the current and projected workforce of nurse practitioners, especially in contrast to the slower growth in physicians, it is not surprising that nurse practitioners are caring for patient populations that are clinically and socially diverse.

Physicians concerned about the appropriate scope for nurse practitioners,^25^,^36^,^45^ have suggested nurse practitioners should focus on providing care for patients with simple, routine, or protocol-based needs. From interviews with primary care nurse practitioners and physicians, prior research found that patients were assigned to providers, either physicians or nurse practitioners, typically by patient preference or provider availability rather than complexity. In the practices interviewed, patient panels for nurse practitioners were typically separate from physicians’ panels (eg, nurse practitioners had “their patients”) with both physicians and nurse practitioners consulting with each other bidirectionally as needed.^31^ Our findings show that nurse practitioners care for patient populations with complex clinical and social needs as often as or more than physicians. The conclusions about the value of this care might differ depending on the alternatives available to patients. Nurse practitioners in rural and poor areas deliver high-quality care in communities that may otherwise lack a clinical provider.^18^ We also found that nurse practitioners were more common in large multispecialty practices than in other types of practices. In those settings, nurse practitioners may deliver team-based, episodic, care collaboratively with physicians.^4^

While our research shows that nurse practitioners care for patients with complex needs, our study does not provide information on the functions of nurse practitioners in care teams.^25^,^37^ In prior surveys of nurse practitioners, ∼40%–65%, reported having their own patient panel^28^,^29^,^33^ which suggests that many nurse practitioners may independently provide care for complex patients. Nurse practitioners and physicians may also jointly manage patients such that they each uniquely contribute to patients’ care and collaboratively share responsibilities based on patient needs and provider expertise.^46^ For example, nurse practitioners caring for patients with complex clinical needs may center their care around disease management, care coordination, or symptom control following defined guidelines.^47^ We found that patients with more chronic conditions had a greater number of visits when cared for by nurse practitioners than physicians. Such care patterns could be consistent with more intense, high-touch care management visits,^24^,^26^ or they could signal the need for more visits to achieve the same goals.

Similarly, practices owned by health systems may have more resources, standardization in care protocols aided by integrated electronic medical records, and sophisticated care management programs which could enable nurse practitioners to better care for patients in these types of practices.^48^,^49^ The fixed cost of such activities may prevent smaller practices from using nurse practitioners in this way.^50^ Further, certain types of care innovations—especially those centered on care delivery, such as advanced care management programs—may be more prevalent in systems.^19^ In small, independent primary care practices, the motivation to use nurse practitioners may be different. The provider shortages faced by practices in underserved areas may lead them to seek nurse practitioners as one way to fill care gaps.

This study has several limitations. First, our analyses of primary care services provided by nurse practitioners versus physicians rely on Medicare billing data, not actual primary care activity. More enhanced clinical data on patterns of care would enhance this study and would strengthen the fit of our models. Nurse practitioners might be providing services which are billed “incident to” the services of the physician with whom they practice; in this case, only the physician’s NPI appears on the claim for that service^51^ therefore, our results may underestimate the role of nurse practitioners in primary care. A 2019 report by the Medicare Payment Advisory Commission (MedPAC) stated that although they could not confidently estimate a precise quantity of “incident to” billing, they estimated that at least some of the services provided by half of the nurse practitioners included “incident to” billing. Second, while some of the growth in the percentage of care provided by nurse practitioners from 2012 to 2017 could be due to shifts in billing practices rather than in actual care patterns,^26^ MedPAC found that the growth in billing by nurse practitioners was consistent with workforce growth.^52^ We used ambulatory visits as a measure of primary care which assumes that all qualifying visits by physicians, such as internists or cardiologists, and nurse practitioners, were for services that could be considered primary care. We used secondary data sources, including IQVIA OneKey data, to identify practice structural characteristics. OneKey data, while it provides detailed information on the relationships between health care organizations and the providers within those organizations, does not offer information on organizations’ structure, governance, or capabilities. Finally, it is not clear how representative this study may be to other US populations such as those covered by Medicaid or commercial payers. While there could be a concern that nurse practitioners care for more clinically complex Medicare beneficiaries because of lower reimbursement rates, research shows that Medicare beneficiaries have high levels of access to care with over 90% of primary care clinicians accepting Medicare.^53^ Further, given Medicare beneficiaries are likely to be sicker and more clinically complex than their commercial counterparts, coupled with an increasingly aging US population, Medicare beneficiaries may be an ideal population for understanding the role of nurse practitioners in caring for complex patients in a rapidly aging population.

It is likely that the role of nurse practitioners will continue to expand given that patient populations are becoming more clinically complex while, at the same time, there is mounting pressure on providers to deliver care that is comprehensive, coordinated, engaged, and cost-effective.^1^,^2^,^10^ Nurse practitioners may provide patient care that is intensive and coordinated across the health care system which could especially benefit patients with complex clinical needs.^24^,^47^ Increasing the role of nurse practitioners in some settings—including small physician practices or practices not owned by a larger health care system—could ease the burden on primary care. Given the increasing role of nurse practitioners, even for clinically and socially complex patients, provider groups may benefit from proactively offering guidance or continuing medical education for practices on optimal workflows to integrating nurse practitioners.

## CONCLUSIONS

Nurse practitioners are a growing part of care delivery for Medicare beneficiaries. Nurse practitioners care for clinically and socially complex patients and they are increasingly delivering care in health care systems. Care delivery reform efforts could learn from the growing presence of nurse practitioners in systems to see how they integrate the services of other clinical providers into care delivery, and also to identify how or whether systems engage nurse practitioners in decision making and leadership around care delivery, an increasingly team-based endeavor.

## Supplementary Material

SUPPLEMENTARY MATERIAL

Supplemental Digital Content is available for this article. Direct URL citations appear in the printed text and are provided in the HTML and PDF versions of this article on the journal's website, www.lww-medicalcare.com.
