# Supplementary material for: Role of Nurse Practitioners in Caring for Patients With Complex Health Needs
Source: Med Care. 2020 Sep 10;58(10):853–60. doi: 10.1097/MLR.0000000000001364 (PMC7552908; doi:10.1097/MLR.0000000000001364)
Supplement: SUPPLEMENTARY MATERIAL [file mlr-58-853-s001.docx]

**SUPPLEMENTAL ONLINE APPENDIX**

[Cohort](#Cohort)

[Practice exclusions](#Practice_exclusions)

[Statistical programs](#Statistical_programs)

[Table 1: Qualifying evaluation and management codes for attribution](#Table_1)

[Table 2: Measure definitions](#Table_2)

[Table 3: Model for count of hierarchical condition categories, 2017 (Figure 2)](#Table_3)

[Table 4: Unadjusted means for hospital utilization, 2017](#Table_4)

[Table 5: Acute care hospital admissions, 2017](#Table_5)

[Table 6: Emergency department visits discharged to home, 2017](#Table_6)

[Table 7: Admissions for ambulatory sensitive conditions, 2017](#Table_7)

[Table 8: Inpatient observation stays, 2017](#Table_8)

[Table 9: Total payments, 2017](#Table_9)

[Table 10: Payments for acute care hospital admissions, 2017](#Table_10)

[Table 11: Other inpatient payments, 2017](#Table_11_new)

[Table 12: All other payments, 2017](#Table_11)

[Table 13: Unadjusted means for ambulatory visits, 2017 (Figure 3)](#Table_12)

[Table 14: Total number of visits, 2017 (Figure 3)](#Table_13)

[Table 15: Number of visits to assigned provider, 2017 (Figure 3)](#Table_14)

[Table 16: Number of visits to a physician, 2017 (Figure 3)](#Table_15)

[Table 17: Number of visits to a nurse practitioner, 2017 (Figure 3)](#Table_16)

[Table 18: Number of visits to a specialist, 2017 (Figure 3)](#Table_17)

[Table 19: Number of providers seen, 2017 (Figure 3)](#Table_18)

[Table 20: Percentage of beneficiaries who get the plurality of their care from a nurse practitioner by state, 2017](#Table_19)

[Table 21: Correlation matrix](#Table_20)

**Cohort**: We assigned beneficiaries to the provider from whom they received the plurality of qualifying evaluation and management visits from outpatient settings, elective teaching hospitals, critical access hospitals, rural health clinics, and federally qualified health clinics. Qualifying evaluation and management visits were based on CMS’ methodology for Medicare Shared Savings Program.^1,2^

**Practice exclusions**: For analyses in Figure 4, we defined practices using IQVIA’s OneKey database as a single site location. After attributing beneficiaries to practices, for Figure 4 analyses, we excluded practices with 125 or fewer attributed beneficiaries. CMS requires cells based on 11 or fewer beneficiaries to be suppressed. To meet CMS’ requirements, when there were between 1 and 11 beneficiaries in a practice with a nurse practitioner as their predominant provider: 1) set the practice to missing when there was more than 1% of beneficiaries attributed to a nurse practitioner and 2) set the practice to have 0% of beneficiaries attributed to nurse practitioners when there was greater than 1% of beneficiaries attributed to nurse practitioners. This suppression approach met CMS’ requirements without skewing the data.

**Statistical programs**: We used SAS for claims extracts and data builds (e.g., create analytic files, compute claims measures, link IQVIA data). Analyses were completed using Stata 16.

**Table 1: Qualifying evaluation and management codes for attribution**

| **HCPCs code** | **Description** |
| --- | --- |
| 99201–99205 | New patient, office, or other outpatient visit |
| 99211–99215 | Established patient, office, or other outpatient visit |
| 99304–99306 | New patient, nursing facility care |
| 99307–99310 | Established patient, nursing facility care |
| 99315–99316 | Established patient, discharge day management service |
| 99318 | Established patient, other nursing facility service |
| 99324–99328 | New patient, domiciliary or rest home visit |
| 99334–99337 | Established patient, domiciliary or rest home visit |
| 99339–99340 | Established patient, physician supervision of patient (patient not present) in home, domiciliary, or rest home |
| 99341–99345 | New patient, home visit |
| 99347–99350 | Established patient, home visit |
| G0402 | Initial Medicare visit |
| G0438-G0439 | Annual wellness visit, initial or subsequent |
| G0463 | Hospital outpatient clinic visit (Electing Teaching Amendment hospitals only) |

**Table 2: Measure definitions**

| **Measure Name** | **Description** |
| --- | --- |
| Primary care physicians | Family practice, general practitioner, internal medicine, geriatrician. Defined using MD-PPAS |
| Nurse practitioner | Defined using MD-PPAS |
| Specialist physicians | Any physician (MD/DO) who is not a primary care physician |
| **Demographics** |  |
| Age | Integer age at beginning of year |
| Median household income | Median household income for beneficiary ZIP Code using US Census Bureau's American Community Survey |
| Residents under poverty level | Percent of population under the federal povery level by census track. Defined by linking beneficiary ZIP Code with US Census Bureau's American Community Survey. |
| Race | Defined using enhanced RTI race categories |
| Disabled | Indicates beneficiary's original reason for Medicare entitlement due to disability |
| Dual eligible for Medicaid | Indicate dual eligibility for Medicare/Medicaid during year |
| Urbanicity | Defined using rural urban commuting codes (RUCA) as isolated rural, small town, micropolitan, or metropolitan. isolated rural, small town, and micropolitan are typically considered "rural." |
| Died | Died in the calendar year |
| **Clinical Characteristics** |  |
| Hierarchical condition categories | Defined using CMS-HCC grouper v22 |
| Coronary artery disease | Derived from HCCs 86, 87, or 88 |
| Congestive heart failure | Derived from HCCs 85 |
| Diabetes | Derived from HCCs 17, 18, 19, or 122 |
| Cancer | Derived from HCCs 8, 9, 10, 11, 12 |
| Chronic obstructive pulmonary disease | Derived from HCC 111 |
| End-stage renal disease | Indicator derived from the denominator file |
| **Inpatient and Outpatient Utilization** |  |
| Inpatient stays | Number of admissions/inpatient stays to acute care/critical access hospitals per beneficiary |
| Emergency department visits discharged to home | Number of emergency department visits from outpatient claims and/or MEDPAR claims per beneficiary |
| Discharges for ambulatory sensitive conditions | Defined using Agency for Healthcare Quality and Research's Prevention Quality Indicators, overall composite |
| Inpatient observation stays | Number of inpatient observational stays from outpatient claims based on revenue center code and CPT code (one visit per date of service) |
| **Payment Categories** |  |
| Total payments | Defined using Berenson-Eggers Type of Services (BETOS) codes. Sum of all codes. |
| Acute care hospital payments | Defined using Berenson-Eggers Type of Services (BETOS) codes. |
| Other inpatient payments | Defined using Berenson-Eggers Type of Services (BETOS) codes. Combined long term care and skilled nursing facility payments |
| Other payments | Defined using Berenson-Eggers Type of Services (BETOS) codes. Durable medical equipment, imaging, evaluation and management visits, procedures, tests, outpatient facilities, home health agency, hospice, and other/exceptions/unclassified payments |

**Table 3: Model for count of hierarchical condition categories, 2017 (Figure 2)**

| R-squared | | 0.270 | | | | | |  |
| --- | --- | --- | --- | --- | --- | --- | --- | --- |
| Number of observations | | 21,039,399 | | | | | |  |
|  | | **Coeff** | | **SE** | **P-value** | **95% LL** | **95% UL** | |
| Nurse practitioner vs. physician | | 0.121 | | 0.001 | 0.000 | 0.119 | 0.123 | |
| Mean age, years | | 0.034 | | 0.000 | 0.000 | 0.033 | 0.034 | |
| Over 85 | | -0.045 | | 0.001 | 0.000 | -0.048 | -0.043 | |
| Under 65 | | 0.251 | | 0.001 | 0.000 | 0.248 | 0.254 | |
| Median household income | | 0.000 | | 0.000 | 0.000 | 0.000 | 0.000 | |
| Residents under poverty level | | -0.001 | | 0.000 | 0.000 | -0.001 | -0.001 | |
| Female | | -0.195 | | 0.001 | 0.000 | -0.196 | -0.194 | |
| Black, non-Hispanic race vs. white, non-Hispanic | | 0.116 | | 0.001 | 0.000 | 0.114 | 0.118 | |
| Hispanic ethnicity vs. white, non-Hispanic | | -0.030 | | 0.001 | 0.000 | -0.033 | -0.028 | |
| Other, non-Hispanic vs. white, non-Hispanic | | -0.128 | | 0.002 | 0.000 | -0.132 | -0.125 | |
| Disabled (original reason for Medicare eligibility) | | 0.601 | | 0.001 | 0.000 | 0.599 | 0.603 | |
| Dual eligible for Medicaid | | 0.451 | | 0.001 | 0.000 | 0.449 | 0.452 | |
| Small town vs. isolated rural | | 0.034 | | 0.002 | 0.000 | 0.031 | 0.037 | |
| Micropolitan vs. isolated rural | | 0.065 | | 0.002 | 0.000 | 0.062 | 0.068 | |
| Metropolitan vs. isolated rural | | 0.111 | | 0.002 | 0.000 | 0.108 | 0.114 | |
| Hospital referral region (suppressed) | |  | |  |  |  |  | |
| Constant | | -2.195 | | 0.005 | 0.000 | -2.204 | -2.186 | |
| **HCC Category** | **Provider Type** | | **Adjusted Mean** | **SE** |  |  |  |  |
| 0 HCCs | Physician | | 40.6% | 0.000 |  |  |  |  |
|  | Nurse practitioner | | 36.2% | 0.000 |  |  |  |  |
| 1-2 HCCs | Physician | | 38.6% | 0.000 |  |  |  |  |
|  | Nurse practitioner | | 37.9% | 0.000 |  |  |  |  |
| 3-5 HCCs | Physician | | 15.9% | 0.000 |  |  |  |  |
|  | Nurse practitioner | | 18.4% | 0.000 |  |  |  |  |
| 6+ HCCs | Physician | | 4.9% | 0.000 |  |  |  |  |
|  | Nurse practitioner | | 7.6% | 0.000 |  |  |  |  |
| **P-value for difference between nurse practitioner vs. physician** | | | F=14.238.62 | P=0.000 |  |  |  |  |

**Table 4: Unadjusted and adjusted means for hospital utilization, 2017**

|  | **Unadjusted** | | **Adjusted** | |
| --- | --- | --- | --- | --- |
|  | **Nurse practitioner** | **Physician** | **Nurse practitioner** | **Physician** |
| Mean number of inpatient stays | 0.393 | 0.280 | .286 | 0.292 |
| Mean number ED visits, discharged home | 0.562 | 0.427 | 0.443 | 0.444 |
| Mean number of potentially avoidable inpatient stays, composite | 0.075 | 0.049 | .052 | .052 |
| Mean number of observation stays | 0.073 | 0.063 | 0.060 | 0.066 |
| Total payments | $12,862 | $9,970 | $10,644 | $10,145 |
| Payments for acute care hospital admissions | $4,254 | $3,128 | $3,016 | $3,226 |
| Other inpatient payments | $2,173 | $930 | $1,667 | $970 |
| All other payments | $6,435 | $5,912 | $5,960 | $5,950 |

**Table 5: Acute care hospital admissions, 2017**

| R2 | 0.4886 |  |  |  |  |  |
| --- | --- | --- | --- | --- | --- | --- |
| Number of observations | 21,039,399 |  |  |  |  |  |
|  | **Coef** | **SE** | **t** | **P-value** | **LL CI** | **UL CI** |
| Nurse practitioner vs. physician | -0.005 | 0.000 | -11.940 | 0.000 | -0.006 | -0.004 |
| Age | -0.003 | 0.000 | -138.860 | 0.000 | -0.003 | -0.003 |
| Median household income | 0.000 | 0.000 | 6.610 | 0.000 | 0.000 | 0.000 |
| Residents under poverty level | 0.000 | 0.000 | 17.460 | 0.000 | 0.000 | 0.000 |
| Number of hierarchical condition categories | 0.294 | 0.000 | 2975.900 | 0.000 | 0.294 | 0.294 |
| Over 85 | 0.014 | 0.001 | 26.960 | 0.000 | 0.013 | 0.015 |
| Under 65 | -0.031 | 0.001 | -45.140 | 0.000 | -0.032 | -0.029 |
| Female | 0.028 | 0.000 | 112.500 | 0.000 | 0.028 | 0.029 |
| Black, non-Hispanic race vs. white, non-Hispanic | 0.015 | 0.000 | 32.340 | 0.000 | 0.014 | 0.016 |
| Hispanic ethnicity vs. white, non-Hispanic | 0.029 | 0.001 | 46.790 | 0.000 | 0.028 | 0.030 |
| Other, non-Hispanic vs. white, non-Hispanic | 0.017 | 0.001 | 24.440 | 0.000 | 0.015 | 0.018 |
| Disabled (original reason for Medicare eligibility) | -0.060 | 0.000 | -127.110 | 0.000 | -0.061 | -0.059 |
| Dual eligible for Medicaid | -0.031 | 0.000 | -83.390 | 0.000 | -0.032 | -0.031 |
| Died in the year | 0.186 | 0.001 | 277.860 | 0.000 | 0.185 | 0.187 |
| Small town vs. isolated rural | -0.008 | 0.001 | -10.650 | 0.000 | -0.010 | -0.007 |
| Micropolitan vs. isolated rural | -0.023 | 0.001 | -32.410 | 0.000 | -0.024 | -0.021 |
| Metropolitan vs. isolated rural | -0.032 | 0.001 | -49.780 | 0.000 | -0.034 | -0.031 |
| Coronary artery disease | 0.062 | 0.001 | 100.510 | 0.000 | 0.061 | 0.063 |
| Congestive heart failure | -0.036 | 0.001 | -70.890 | 0.000 | -0.037 | -0.035 |
| Diabetes | -0.272 | 0.000 | -838.580 | 0.000 | -0.273 | -0.272 |
| Cancer | -0.166 | 0.000 | -370.360 | 0.000 | -0.167 | -0.165 |
| Chronic obstructive pulmonary disease | -0.089 | 0.000 | -194.560 | 0.000 | -0.090 | -0.088 |
| End stage renal disease | -0.026 | 0.001 | -22.170 | 0.000 | -0.029 | -0.024 |
| Hospital referral region (suppressed) |  |  |  |  |  |  |
| Constant | 0.182 | 0.002 | 86.260 | 0.000 | 0.177 | 0.186 |
| **Adjusted Means** | **Margin** | **SE** | **Z** | **P-value** | **LL CI** | **UL CI** |
| Physician | 0.292 | 0.000 | 2247.760 | 0.000 | 0.292 | 0.292 |
| Nurse practitioner | 0.286 | 0.000 | 666.670 | 0.000 | 0.286 | 0.287 |
| **P-value for the difference between nurse practitioners and physicians** | F=142.49 | P<0.000 |  |  |  |  |

**Table 6: Emergency department visits discharged to home, 2017**

| R2 | 0.089 |  |  |  |  |  |
| --- | --- | --- | --- | --- | --- | --- |
| Number of observations | 21,039,399 |  |  |  |  |  |
|  | **Coef** | **SE** | **t** | **P-value** | **LL CI** | **UL CI** |
| Nurse practitioner vs. physician | -0.001 | 0.001 | -1.050 | 0.292 | -0.003 | 0.001 |
| Age | -0.003 | 0.000 | -82.040 | 0.000 | -0.003 | -0.003 |
| Median household income | 0.000 | 0.000 | 3.090 | 0.002 | 0.000 | 0.000 |
| Residents under poverty level | 0.001 | 0.000 | 23.190 | 0.000 | 0.001 | 0.001 |
| Number of hierarchical condition categories | 0.150 | 0.000 | 743.030 | 0.000 | 0.149 | 0.150 |
| Over 85 | 0.173 | 0.001 | 160.000 | 0.000 | 0.171 | 0.175 |
| Under 65 | 0.188 | 0.001 | 134.830 | 0.000 | 0.185 | 0.190 |
| Female | 0.065 | 0.001 | 126.560 | 0.000 | 0.064 | 0.066 |
| Black, non-Hispanic race vs. white, non-Hispanic | 0.132 | 0.001 | 135.880 | 0.000 | 0.130 | 0.134 |
| Hispanic ethnicity vs. white, non-Hispanic | 0.019 | 0.001 | 14.840 | 0.000 | 0.016 | 0.021 |
| Other, non-Hispanic vs. white, non-Hispanic | -0.090 | 0.001 | -64.460 | 0.000 | -0.092 | -0.087 |
| Disabled (original reason for Medicare eligibility) | 0.075 | 0.001 | 77.520 | 0.000 | 0.073 | 0.077 |
| Dual eligible for Medicaid | 0.149 | 0.001 | 193.760 | 0.000 | 0.148 | 0.151 |
| Died in the year | -0.212 | 0.001 | 155.140 | 0.000 | -0.215 | -0.209 |
| Small town vs. isolated rural | 0.044 | 0.002 | 27.740 | 0.000 | 0.041 | 0.047 |
| Micropolitan vs. isolated rural | 0.009 | 0.001 | 5.950 | 0.000 | 0.006 | 0.011 |
| Metropolitan vs. isolated rural | -0.070 | 0.001 | -52.750 | 0.000 | -0.073 | -0.068 |
| Coronary artery disease | 0.084 | 0.001 | 66.340 | 0.000 | 0.081 | 0.086 |
| Congestive heart failure | -0.040 | 0.001 | -38.530 | 0.000 | -0.042 | -0.038 |
| Diabetes | -0.091 | 0.001 | 137.500 | 0.000 | -0.092 | -0.090 |
| Cancer | -0.084 | 0.001 | -92.290 | 0.000 | -0.086 | -0.083 |
| Chronic obstructive pulmonary disease | 0.074 | 0.001 | 79.510 | 0.000 | 0.073 | 0.076 |
| End stage renal disease | 0.015 | 0.002 | 6.050 | 0.000 | 0.010 | 0.019 |
| Hospital referral region (suppressed) |  |  |  |  |  |  |
| Constant | 0.366 | 0.004 | 85.180 | 0.000 | 0.357 | 0.374 |
| **Adjusted Means** | **Margin** | **SE** | **Z** | **P-value** | **LL CI** | **UL CI** |
| Physician | 0.444 | 0.000 | 1677.350 | 0.000 | 0.444 | 0.445 |
| Nurse practitioner | 0.443 | 0.001 | 505.720 | 0.000 | 0.442 | 0.445 |
| **P-value for the difference between nurse practitioners and physicians** | F=1.11 | P=.2924 |  |  |  |  |

**Table 7: Admissions for ambulatory sensitive conditions, 2017**

| R2 | 0.165 |  |  |  |  |  |
| --- | --- | --- | --- | --- | --- | --- |
| Number of observations | 21,039,399 |  |  |  |  |  |
|  | **Coef** | **SE** | **t** | **P-value** | **LL CI** | **UL CI** |
| Nurse practitioner vs. physician | 0.000 | 0.000 | 0.020 | 0.982 | 0.000 | 0.000 |
| Age | 0.000 | 0.000 | -19.260 | 0.000 | 0.000 | 0.000 |
| Median household income | 0.000 | 0.000 | 2.760 | 0.006 | 0.000 | 0.000 |
| Residents under poverty level | 0.000 | 0.000 | 14.210 | 0.000 | 0.000 | 0.000 |
| Number of hierarchical condition categories | 0.040 | 0.000 | 848.590 | 0.000 | 0.040 | 0.040 |
| Over 85 | 0.015 | 0.000 | 59.490 | 0.000 | 0.014 | 0.015 |
| Under 65 | -0.003 | 0.000 | -9.850 | 0.000 | -0.004 | -0.003 |
| Female | 0.010 | 0.000 | 80.180 | 0.000 | 0.009 | 0.010 |
| Black, non-Hispanic race vs. white, non-Hispanic | 0.013 | 0.000 | 55.470 | 0.000 | 0.012 | 0.013 |
| Hispanic ethnicity vs. white, non-Hispanic | 0.008 | 0.000 | 28.630 | 0.000 | 0.008 | 0.009 |
| Other, non-Hispanic vs. white, non-Hispanic | 0.004 | 0.000 | 11.150 | 0.000 | 0.003 | 0.004 |
| Disabled (original reason for Medicare eligibility) | -0.007 | 0.000 | -30.060 | 0.000 | -0.007 | -0.006 |
| Dual eligible for Medicaid | 0.000 | 0.000 | -2.470 | 0.013 | -0.001 | 0.000 |
| Died in the year | 0.040 | 0.000 | 125.480 | 0.000 | 0.039 | 0.040 |
| Small town vs. isolated rural | -0.002 | 0.000 | -6.540 | 0.000 | -0.003 | -0.002 |
| Micropolitan vs. isolated rural | -0.007 | 0.000 | -22.440 | 0.000 | -0.008 | -0.007 |
| Metropolitan vs. isolated rural | -0.008 | 0.000 | -26.370 | 0.000 | -0.009 | -0.008 |
| Coronary artery disease | 0.008 | 0.000 | 28.670 | 0.000 | 0.008 | 0.009 |
| Congestive heart failure | 0.124 | 0.000 | 515.140 | 0.000 | 0.124 | 0.124 |
| Diabetes | -0.017 | 0.000 | -113.460 | 0.000 | -0.018 | -0.017 |
| Cancer | -0.046 | 0.000 | -216.760 | 0.000 | -0.046 | -0.046 |
| Chronic obstructive pulmonary disease | 0.084 | 0.000 | 386.660 | 0.000 | 0.083 | 0.084 |
| End stage renal disease | -0.012 | 0.001 | -20.760 | 0.000 | -0.013 | -0.011 |
| Hospital referral region (suppressed) |  |  |  |  |  |  |
| Constant | -0.005 | 0.001 | -5.420 | 0.000 | -0.007 | -0.003 |
| **Adjusted Means** | **Margin** | **SE** | **Z** | **P-value** | **LL CI** | **UL CI** |
| Physician | 0.052 | 0.000 | 838.980 | 0.000 | 0.051 | 0.052 |
| Nurse practitioner | 0.052 | 0.000 | 253.530 | 0.000 | 0.051 | 0.052 |
| **P-value for the difference between nurse practitioners and physicians** | F=0 | P=.9819 |  |  |  |  |

**Table 8: Inpatient observation stays, 2017**

| R2 | 0.037 |  |  |  |  |  |
| --- | --- | --- | --- | --- | --- | --- |
| Number of observations | 21,039,399 |  |  |  |  |  |
|  | **Coef** | **SE** | **t** | **P-value** | **LL CI** | **UL CI** |
| Nurse practitioner vs. physician | -0.006 | 0.000 | -20.840 | 0.000 | -0.006 | -0.005 |
| Age | 0.001 | 0.000 | 59.630 | 0.000 | 0.001 | 0.001 |
| Median household income | 0.000 | 0.000 | 6.420 | 0.000 | 0.000 | 0.000 |
| Residents under poverty level | 0.000 | 0.000 | 12.430 | 0.000 | 0.000 | 0.000 |
| Number of hierarchical condition categories | 0.024 | 0.000 | 397.800 | 0.000 | 0.024 | 0.024 |
| Over 85 | 0.012 | 0.000 | 38.230 | 0.000 | 0.012 | 0.013 |
| Under 65 | 0.014 | 0.000 | 33.140 | 0.000 | 0.013 | 0.015 |
| Female | 0.008 | 0.000 | 55.120 | 0.000 | 0.008 | 0.009 |
| Black, non-Hispanic race vs. white, non-Hispanic | 0.010 | 0.000 | 35.750 | 0.000 | 0.010 | 0.011 |
| Hispanic ethnicity vs. white, non-Hispanic | 0.001 | 0.000 | 3.470 | 0.001 | 0.001 | 0.002 |
| Other, non-Hispanic vs. white, non-Hispanic | -0.008 | 0.000 | -18.100 | 0.000 | -0.008 | -0.007 |
| Disabled (original reason for Medicare eligibility) | 0.011 | 0.000 | 37.660 | 0.000 | 0.010 | 0.011 |
| Dual eligible for Medicaid | 0.004 | 0.000 | 19.380 | 0.000 | 0.004 | 0.005 |
| Died in the year | -0.046 | 0.000 | -113.220 | 0.000 | -0.047 | -0.045 |
| Small town vs. isolated rural | -0.004 | 0.000 | -7.500 | 0.000 | -0.004 | -0.003 |
| Micropolitan vs. isolated rural | -0.009 | 0.000 | -20.460 | 0.000 | -0.010 | -0.008 |
| Metropolitan vs. isolated rural | -0.017 | 0.000 | -41.700 | 0.000 | -0.017 | -0.016 |
| Coronary artery disease | 0.093 | 0.000 | 247.020 | 0.000 | 0.092 | 0.094 |
| Congestive heart failure | 0.017 | 0.000 | 54.410 | 0.000 | 0.016 | 0.017 |
| Diabetes | -0.011 | 0.000 | -57.880 | 0.000 | -0.012 | -0.011 |
| Cancer | -0.004 | 0.000 | -15.530 | 0.000 | -0.005 | -0.004 |
| Chronic obstructive pulmonary disease | 0.012 | 0.000 | 41.500 | 0.000 | 0.011 | 0.012 |
| End stage renal disease | 0.034 | 0.001 | 47.150 | 0.000 | 0.033 | 0.036 |
| Hospital referral region (suppressed) |  |  |  |  |  |  |
| Constant | -0.048 | 0.001 | -37.680 | 0.000 | -0.051 | -0.046 |
| **Adjusted Means** | **Margin** | **SE** | **Z** | **P-value** | **LL CI** | **UL CI** |
| Physician | 0.066 | 0.000 | 829.270 | 0.000 | 0.065 | 0.066 |
| Nurse practitioner | 0.060 | 0.000 | 228.720 | 0.000 | 0.059 | 0.060 |
| **P-value for the difference between nurse practitioners and physicians** | F=434.42 | P>F=0.000 |  |  |  |  |

**Table 9: Total payments, 2017**

| R2 | 0.456 |  |  |  |  |  |
| --- | --- | --- | --- | --- | --- | --- |
| Number of observations | 21,039,399 |  |  |  |  |  |
|  | **Coef** | **SE** | **t** | **P-value** | **LL CI** | **UL CI** |
| Nurse practitioner vs. physician | 498.36 | 13.053 | 38.180 | 0.000 | 472.78 | 523.94 |
| Age | -36.50 | 0.583 | -62.560 | 0.000 | -37.64 | -35.35 |
| Median household income | 0.00 | 0.000 | 18.300 | 0.000 | 0.00 | 0.00 |
| Residents under poverty level | 4.61 | 0.515 | 8.950 | 0.000 | 3.60 | 5.62 |
| Number of hierarchical condition categories | 7971.10 | 2.865 | 2782.170 | 0.000 | 7965.49 | 7976.72 |
| Over 85 | -270.74 | 15.387 | -17.600 | 0.000 | -300.90 | -240.58 |
| Under 65 | -1383.75 | 19.762 | -70.020 | 0.000 | -1422.49 | -1345.02 |
| Female | 727.43 | 7.331 | 99.230 | 0.000 | 713.06 | 741.80 |
| Black, non-Hispanic race vs. white, non-Hispanic | -58.76 | 13.829 | -4.250 | 0.000 | -85.86 | -31.66 |
| Hispanic ethnicity vs. white, non-Hispanic | 22.11 | 18.027 | 1.230 | 0.220 | -13.22 | 57.44 |
| Other, non-Hispanic vs. white, non-Hispanic | -479.58 | 19.751 | -24.280 | 0.000 | -518.30 | -440.87 |
| Disabled (original reason for Medicare eligibility) | -942.32 | 13.749 | -68.540 | 0.000 | -969.27 | -915.37 |
| Dual eligible for Medicaid | -672.49 | 10.943 | -61.460 | 0.000 | -693.93 | -651.04 |
| Died in the year | 2557.87 | 19.413 | 131.760 | 0.000 | 2519.82 | 2595.92 |
| Small town vs. isolated rural | -250.98 | 22.380 | -11.210 | 0.000 | -294.85 | -207.12 |
| Micropolitan vs. isolated rural | -808.93 | 20.428 | -39.600 | 0.000 | -848.97 | -768.89 |
| Metropolitan vs. isolated rural | -943.21 | 18.881 | -49.950 | 0.000 | -980.22 | -906.21 |
| Coronary artery disease | -338.77 | 17.945 | -18.880 | 0.000 | -373.94 | -303.60 |
| Congestive heart failure | -3804.13 | 14.755 | -257.820 | 0.000 | -3833.05 | -3775.21 |
| Diabetes | -7341.90 | 9.416 | -779.740 | 0.000 | -7360.36 | -7323.45 |
| Cancer | 497.84 | 12.999 | 38.300 | 0.000 | 472.36 | 523.32 |
| Chronic obstructive pulmonary disease | -5256.76 | 13.290 | -395.540 | 0.000 | -5282.81 | -5230.71 |
| End stage renal disease | 20140.34 | 34.538 | 583.130 | 0.000 | 20072.65 | 20208.04 |
| Hospital referral region (suppressed) |  |  |  |  |  |  |
| Constant | 3181.30 | 61.014 | 52.140 | 0.000 | 3061.72 | 3300.89 |
| **Adjusted Means** | **Margin** | **SE** | **Z** | **P-value** | **LL CI** | **UL CI** |
| Physician | 10145.48 | 3.762 | 2696.68 | 0.000 | 10138.11 | 10152.86 |
| Nurse practitioner | 10643.85 | 12.451 | 854.86 | 0.000 | 10619.44 | 10668.25 |
| **P-value for the difference between nurse practitioners and physicians** | F=1457.75 | P<0.000 |  |  |  |  |

**Table 10: Payments for acute care hospital admissions, 2017**

| R2 | 0.373 |  |  |  |  |  |
| --- | --- | --- | --- | --- | --- | --- |
| Number of observations | 21,039,399 |  |  |  |  |  |
|  | **Coef** | **SE** | **t** | **P-value** | **LL CI** | **UL CI** |
| Nurse practitioner vs. physician | -209.92 | 7.473 | -28.090 | 0.000 | -224.56 | -195.27 |
| Age | -62.32 | 0.334 | -186.590 | 0.000 | -62.98 | -61.67 |
| Median household income | 0.00 | 0.000 | -1.640 | 0.101 | 0.00 | 0.00 |
| Residents under poverty level | 4.83 | 0.295 | 16.390 | 0.000 | 4.25 | 5.41 |
| Number of hierarchical condition categories | 3992.71 | 1.640 | 2434.120 | 0.000 | 3989.49 | 3995.92 |
| Over 85 | -795.92 | 8.809 | -90.350 | 0.000 | -813.19 | -778.65 |
| Under 65 | -831.23 | 11.314 | -73.470 | 0.000 | -853.41 | -809.06 |
| Female | 96.26 | 4.197 | 22.940 | 0.000 | 88.03 | 104.49 |
| Black, non-Hispanic race vs. white, non-Hispanic | 246.50 | 7.917 | 31.130 | 0.000 | 230.98 | 262.02 |
| Hispanic ethnicity vs. white, non-Hispanic | 473.34 | 10.321 | 45.860 | 0.000 | 453.12 | 493.57 |
| Other, non-Hispanic vs. white, non-Hispanic | 387.14 | 11.308 | 34.240 | 0.000 | 364.98 | 409.31 |
| Disabled (original reason for Medicare eligibility) | -1143.42 | 7.871 | -145.260 | 0.000 | -1158.85 | -1127.99 |
| Dual eligible for Medicaid | -807.64 | 6.265 | -128.920 | 0.000 | -819.92 | -795.36 |
| Died in the year | 3668.44 | 11.114 | 330.070 | 0.000 | 3646.66 | 3690.23 |
| Small town vs. isolated rural | -123.84 | 12.813 | -9.670 | 0.000 | -148.96 | -98.73 |
| Micropolitan vs. isolated rural | -309.45 | 11.695 | -26.460 | 0.000 | -332.37 | -286.53 |
| Metropolitan vs. isolated rural | -498.19 | 10.810 | -46.090 | 0.000 | -519.38 | -477.01 |
| Coronary artery disease | 1326.68 | 10.274 | 129.130 | 0.000 | 1306.54 | 1346.81 |
| Congestive heart failure | -1601.61 | 8.448 | -189.590 | 0.000 | -1618.16 | -1585.05 |
| Diabetes | -4062.02 | 5.391 | -753.520 | 0.000 | -4072.59 | -4051.46 |
| Cancer | -2576.19 | 7.442 | -346.150 | 0.000 | -2590.78 | -2561.60 |
| Chronic obstructive pulmonary disease | -2873.58 | 7.609 | -377.660 | 0.000 | -2888.49 | -2858.66 |
| End stage renal disease | 1924.55 | 19.774 | 97.330 | 0.000 | 1885.80 | 1963.31 |
| Hospital referral region (suppressed) |  |  |  |  |  |  |
| Constant | 3521.35 | 34.931 | 100.810 | 0.000 | 3452.89 | 3589.82 |
| **Adjusted Means** | **Margin** | **SE** | **Z** | **P-value** | **LL CI** | **UL CI** |
| Physician | 3225.99 | 2.154 | 1497.720 | 0.000 | 3221.77 | 3230.21 |
| Nurse practitioner | 3016.08 | 7.128 | 423.100 | 0.000 | 3002.10 | 3030.05 |
| **P-value for the difference between nurse practitioners and physicians** | F=789.05 | P<.0.000 |  |  |  |  |

**Table 11: Other inpatient payments, 2017**

| R2 | 0.176 |  |  |  |  |  |
| --- | --- | --- | --- | --- | --- | --- |
| Number of observations | 21,039,399 |  |  |  |  |  |
|  | **Coef** | **SE** | **t** | **P-value** | **LL CI** | **UL CI** |
| Nurse practitioner vs. physician | 697.36 | 4.440 | 157.060 | 0.000 | 688.65 | 706.06 |
| Age | 4.51 | 0.198 | 22.710 | 0.000 | 4.12 | 4.90 |
| Median household income | 0.00 | 0.000 | -4.020 | 0.000 | 0.00 | 0.00 |
| Residents under poverty level | 1.38 | 0.175 | 7.880 | 0.000 | 1.04 | 1.72 |
| Number of hierarchical condition categories | 1651.59 | 0.975 | 1694.600 | 0.000 | 1649.68 | 1653.50 |
| Over 85 | 320.40 | 5.234 | 61.210 | 0.000 | 310.15 | 330.66 |
| Under 65 | -260.41 | 6.722 | -38.740 | 0.000 | -273.58 | -247.23 |
| Female | 125.34 | 2.494 | 50.260 | 0.000 | 120.45 | 130.22 |
| Black, non-Hispanic race vs. white, non-Hispanic | 112.01 | 4.704 | 23.810 | 0.000 | 102.79 | 121.23 |
| Hispanic ethnicity vs. white, non-Hispanic | -26.64 | 6.132 | -4.340 | 0.000 | -38.66 | -14.62 |
| Other, non-Hispanic vs. white, non-Hispanic | 4.39 | 6.719 | 0.650 | 0.513 | -8.78 | 17.56 |
| Disabled (original reason for Medicare eligibility) | -276.29 | 4.677 | -59.070 | 0.000 | -285.45 | -267.12 |
| Dual eligible for Medicaid | -8.24 | 3.722 | -2.210 | 0.027 | -15.54 | -0.95 |
| Died in the year | -443.69 | 6.604 | -67.190 | 0.000 | -456.63 | -430.74 |
| Small town vs. isolated rural | -87.86 | 7.613 | -11.540 | 0.000 | -102.78 | -72.94 |
| Micropolitan vs. isolated rural | -255.27 | 6.949 | -36.730 | 0.000 | -268.89 | -241.65 |
| Metropolitan vs. isolated rural | -246.16 | 6.423 | -38.330 | 0.000 | -258.75 | -233.57 |
| Coronary artery disease | -1468.04 | 6.104 | -240.490 | 0.000 | -1480.00 | -1456.07 |
| Congestive heart failure | -1343.89 | 5.019 | -267.740 | 0.000 | -1353.73 | -1334.05 |
| Diabetes | -1624.99 | 3.203 | -507.330 | 0.000 | -1631.27 | -1618.71 |
| Cancer | -1934.95 | 4.422 | -437.570 | 0.000 | -1943.61 | -1926.28 |
| Chronic obstructive pulmonary disease | -1403.32 | 4.521 | -310.410 | 0.000 | -1412.18 | -1394.46 |
| End stage renal disease | -2529.50 | 11.749 | -215.300 | 0.000 | -2552.52 | -2506.47 |
| Hospital referral region (suppressed) |  |  |  |  |  |  |
| Constant | -692.77 | 20.755 | -33.380 | 0.000 | -733.45 | -652.09 |
| **Adjusted Means** | **Margin** | **SE** | **Z** | **P-value** | **LL CI** | **UL CI** |
| Physician | 969.97 | 1.280 | 757.910 | 0.000 | 967.46 | 972.48 |
| Nurse practitioner | 1667.33 | 4.235 | 393.660 | 0.000 | 1659.02 | 1675.63 |
| **P-value for the difference between nurse practitioners and physicians** | F=2466.52 | P=0.000 |  |  |  |  |

**Table 12: All other payments, 2017**

| R2 | 0.254 |  |  |  |  |  |
| --- | --- | --- | --- | --- | --- | --- |
| Number of observations | 21,039,399 |  |  |  |  |  |
|  | **Coef** | **SE** | **t** | **P-value** | **LL CI** | **UL CI** |
| Nurse practitioner vs. physician | 10.92 | 7.975 | 1.370 | 0.171 | -4.71 | 26.55 |
| Age | 21.32 | 0.356 | 59.810 | 0.000 | 20.62 | 22.02 |
| Median household income | 0.00 | 0.000 | 33.730 | 0.000 | 0.00 | 0.00 |
| Residents under poverty level | -1.60 | 0.315 | -5.100 | 0.000 | -2.22 | -0.99 |
| Number of hierarchical condition categories | 2326.81 | 1.751 | 1329.190 | 0.000 | 2323.38 | 2330.24 |
| Over 85 | 204.78 | 9.401 | 21.780 | 0.000 | 186.35 | 223.20 |
| Under 65 | -292.11 | 12.074 | -24.190 | 0.000 | -315.78 | -268.45 |
| Female | 505.83 | 4.479 | 112.940 | 0.000 | 497.06 | 514.61 |
| Black, non-Hispanic race vs. white, non-Hispanic | -417.27 | 8.449 | -49.380 | 0.000 | -433.83 | -400.71 |
| Hispanic ethnicity vs. white, non-Hispanic | -424.60 | 11.015 | -38.550 | 0.000 | -446.18 | -403.01 |
| Other, non-Hispanic vs. white, non-Hispanic | -871.12 | 12.068 | -72.190 | 0.000 | -894.77 | -847.47 |
| Disabled (original reason for Medicare eligibility) | 477.39 | 8.400 | 56.830 | 0.000 | 460.92 | 493.85 |
| Dual eligible for Medicaid | 143.40 | 6.686 | 21.450 | 0.000 | 130.29 | 156.50 |
| Died in the year | -666.89 | 11.861 | -56.220 | 0.000 | -690.13 | -643.64 |
| Small town vs. isolated rural | -39.28 | 13.674 | -2.870 | 0.004 | -66.08 | -12.48 |
| Micropolitan vs. isolated rural | -244.21 | 12.481 | -19.570 | 0.000 | -268.68 | -219.75 |
| Metropolitan vs. isolated rural | -198.86 | 11.536 | -17.240 | 0.000 | -221.47 | -176.25 |
| Coronary artery disease | -197.41 | 10.964 | -18.010 | 0.000 | -218.90 | -175.92 |
| Congestive heart failure | -858.64 | 9.015 | -95.240 | 0.000 | -876.31 | -840.97 |
| Diabetes | -1654.89 | 5.753 | -287.650 | 0.000 | -1666.17 | -1643.62 |
| Cancer | 5008.98 | 7.943 | 630.650 | 0.000 | 4993.41 | 5024.55 |
| Chronic obstructive pulmonary disease | -979.87 | 8.120 | -120.670 | 0.000 | -995.78 | -963.95 |
| End stage renal disease | 20745.29 | 21.103 | 983.060 | 0.000 | 20703.93 | 20786.65 |
| Hospital referral region (suppressed) |  |  |  |  |  |  |
| Constant | 352.72 | 37.279 | 9.460 | 0.000 | 279.65 | 425.78 |
| **Adjusted Means** | **Margin** | **SE** | **Z** | **P-value** | **LL CI** | **UL CI** |
| Physician | 5949.53 | 2.299 | 2588.210 | 0.000 | 5945.02 | 5954.03 |
| Nurse practitioner | 5960.45 | 7.608 | 783.490 | 0.000 | 5945.54 | 5975.36 |
| **P-value for the difference between nurse practitioners and physicians** | F=1.88 | P=.1709 |  |  |  |  |

**Table 13: Unadjusted means for ambulatory visits, 2017 (Figure 3)**

| Beneficiary Number of Hierarchical Condition Categories | 0 | | 1-2 | | 3-5 | | 6+ | |
| --- | --- | --- | --- | --- | --- | --- | --- | --- |
|  | **MD** | **NP** | **MD** | **NP** | **MD** | **NP** | **MD** | **NP** |
| Mean number of visits | 6.2 | 5.9 | 11.0 | 11.6 | 16.7 | 18.2 | 20.8 | 24.0 |
| Mean number of visits to a physician | 5.6 | 3.2 | 9.8 | 5.2 | 14.7 | 8.0 | 17.9 | 10.8 |
| Mean number of visits to a specialist | 3.8 | 2.6 | 6.3 | 4.1 | 9.3 | 5.7 | 11.0 | 6.8 |
| Mean number of visits to a nurse practitioner | 1.6 | 3.5 | 2.1 | 6.5 | 2.8 | 9.7 | 3.6 | 12.3 |
| Mean number of providers seen | 3.1 | 2.9 | 4.4 | 4.3 | 5.9 | 5.8 | 6.8 | 7.0 |
| Mean number of visits to assigned provider | 3.2 | 3.1 | 5.1 | 5.6 | 7.0 | 7.9 | 8.3 | 9.6 |
| Mean number of visits to unassigned provider | 3.0 | 2.8 | 5.9 | 6.0 | 9.8 | 10.3 | 12.5 | 14.3 |
| Share of visits to assigned provider | 62.6% | 63.8% | 52.9% | 53.5% | 46.3% | 47.0% | 45.1% | 44.1% |

**Table 14: Total number of visits, 2017 (Figure 3)**

| R-squared | 0.252 |  |  |  |  |
| --- | --- | --- | --- | --- | --- |
| Number of observations | 21,039,399 |  |  |  |  |
|  | **Coef** | **SE** | **P-value** | **LL CI** | **LL CI** |
| Nurse practitioner vs. physician | -0.022 | 0.010 | 0.029 | -0.042 | -0.002 |
| 1 to 2 vs 0 hierarchical condition categories | 4.646 | 0.004 | 0.000 | 4.639 | 4.654 |
| 3 to 5 vs 0 hierarchical condition categories | 10.217 | 0.006 | 0.000 | 10.206 | 10.229 |
| 6+ vs 0 hierarchical condition categories | 14.012 | 0.008 | 0.000 | 13.996 | 14.029 |
| Nurse practitioner vs. physician * hierarchical condition categories |  |  |  |  |  |
| Nurse practitioner*1-2 conditions | 0.799 | 0.014 | 0.000 | 0.772 | 0.827 |
| Nurse practitioner*3-5 conditions | 1.499 | 0.018 | 0.000 | 1.463 | 1.535 |
| Nurse practitioner*6+ conditions | 3.149 | 0.024 | 0.000 | 3.103 | 3.195 |
| Mean age, years | 0.063 | 0.000 | 0.000 | 0.062 | 0.063 |
| Over 85 | -1.460 | 0.007 | 0.000 | -1.475 | -1.446 |
| Under 65 | 0.154 | 0.009 | 0.000 | 0.135 | 0.172 |
| Median household income | 0.000 | 0.000 | 0.000 | 0.000 | 0.000 |
| Residents under poverty level | 0.002 | 0.000 | 0.000 | 0.002 | 0.003 |
| Female | 0.968 | 0.004 | 0.000 | 0.961 | 0.975 |
| Black, non-Hispanic race vs. white, non-Hispanic | -1.221 | 0.007 | 0.000 | -1.234 | -1.208 |
| Hispanic ethnicity vs. white, non-Hispanic | -1.142 | 0.009 | 0.000 | -1.159 | -1.125 |
| Other, non-Hispanic vs. white, non-Hispanic | -1.141 | 0.009 | 0.000 | -1.160 | -1.123 |
| Disabled (original reason for Medicare eligibility) | 0.967 | 0.007 | 0.000 | 0.954 | 0.980 |
| Dual eligible for Medicaid | 0.682 | 0.005 | 0.000 | 0.672 | 0.693 |
| Small town vs. isolated rural | -0.008 | 0.011 | 0.459 | -0.029 | 0.013 |
| Micropolitan vs. isolated rural | 0.330 | 0.010 | 0.000 | 0.311 | 0.349 |
| Metropolitan vs. isolated rural | 0.602 | 0.009 | 0.000 | 0.585 | 0.620 |
| Hospital referral region (suppressed) |  |  |  |  |  |
| Constant | -0.676 | 0.029 | 0.000 | -0.734 | -0.619 |
| **P-value for difference between nurse practitioner vs. physician** | F= 12,254.130 | P=0.000 |  |  |  |

**Table 15: Number of visits to assigned provider, 2017 (Figure 3)**

| R-squared | 0.156 |  |  |  |  |
| --- | --- | --- | --- | --- | --- |
| Number of observations | 21,039,399 |  |  |  |  |
|  | **Coef** | **SE** | **P-value** | **LL CI** | **LL CI** |
| Nurse practitioner vs. physician | -0.138 | 0.005 | 0.000 | -0.148 | -0.128 |
| 1 to 2 vs 0 hierarchical condition categories | 1.742 | 0.002 | 0.000 | 1.738 | 1.746 |
| 3 to 5 vs 0 hierarchical condition categories | 3.428 | 0.003 | 0.000 | 3.422 | 3.434 |
| 6+ vs 0 hierarchical condition categories | 4.561 | 0.004 | 0.000 | 4.553 | 4.569 |
| Nurse practitioner vs. physician * hierarchical condition categories |  |  |  |  |  |
| Nurse practitioner*1-2 conditions | 0.425 | 0.007 | 0.000 | 0.411 | 0.439 |
| Nurse practitioner*3-5 conditions | 0.842 | 0.009 | 0.000 | 0.824 | 0.860 |
| Nurse practitioner*6+ conditions | 1.366 | 0.012 | 0.000 | 1.343 | 1.390 |
| Mean age, years | 0.035 | 0.000 | 0.000 | 0.035 | 0.035 |
| Over 85 | -0.133 | 0.004 | 0.000 | -0.141 | -0.126 |
| Under 65 | 0.265 | 0.005 | 0.000 | 0.256 | 0.275 |
| Median household income | 0.000 | 0.000 | 0.000 | 0.000 | 0.000 |
| Residents under poverty level | 0.003 | 0.000 | 0.000 | 0.002 | 0.003 |
| Female | 0.352 | 0.002 | 0.000 | 0.348 | 0.355 |
| Black, non-Hispanic race vs. white, non-Hispanic | -0.261 | 0.003 | 0.000 | -0.267 | -0.254 |
| Hispanic ethnicity vs. white, non-Hispanic | -0.216 | 0.004 | 0.000 | -0.225 | -0.207 |
| Other, non-Hispanic vs. white, non-Hispanic | -0.064 | 0.005 | 0.000 | -0.073 | -0.055 |
| Disabled (original reason for Medicare eligibility) | 0.468 | 0.003 | 0.000 | 0.462 | 0.475 |
| Dual eligible for Medicaid | 1.092 | 0.003 | 0.000 | 1.086 | 1.097 |
| Small town vs. isolated rural | -0.031 | 0.005 | 0.000 | -0.042 | -0.020 |
| Micropolitan vs. isolated rural | -0.044 | 0.005 | 0.000 | -0.054 | -0.034 |
| Metropolitan vs. isolated rural | -0.176 | 0.005 | 0.000 | -0.185 | -0.167 |
| Hospital referral region (suppressed) |  |  |  |  |  |
| Constant | 0.156 | 0.015 | 0.000 | 0.127 | 0.185 |
| **P-value for difference between nurse practitioner vs. physician** | F=5352.54 | P<.0000 |  |  |  |

**Table 16: Number of visits to a physician, 2017 (Figure 3)**

| R-squared | 0.248 |  |  |  |  |
| --- | --- | --- | --- | --- | --- |
| Number of observations | 20,628,519 |  |  |  |  |
|  | **Coef** | **SE** | **P-value** | **LL CI** | **LL CI** |
| Nurse practitioner vs. physician | -2.030 | 0.011 | 0.000 | -2.051 | -2.008 |
| 1 to 2 vs 0 hierarchical condition categories | 4.139 | 0.004 | 0.000 | 4.132 | 4.146 |
| 3 to 5 vs 0 hierarchical condition categories | 8.946 | 0.005 | 0.000 | 8.936 | 8.955 |
| 6+ vs 0 hierarchical condition categories | 11.977 | 0.007 | 0.000 | 11.963 | 11.992 |
| Nurse practitioner vs. physician * hierarchical condition categories |  |  |  |  |  |
| Nurse practitioner*1-2 conditions | -2.138 | 0.014 | 0.000 | -2.166 | -2.110 |
| Nurse practitioner*3-5 conditions | -4.340 | 0.017 | 0.000 | -4.374 | -4.305 |
| Nurse practitioner*6+ conditions | -4.783 | 0.022 | 0.000 | -4.825 | -4.740 |
| Mean age, years | 0.050 | 0.000 | 0.000 | 0.049 | 0.050 |
| Over 85 | -1.414 | 0.006 | 0.000 | -1.427 | -1.402 |
| Under 65 | 0.210 | 0.008 | 0.000 | 0.194 | 0.226 |
| Median household income | 0.000 | 0.000 | 0.000 | 0.000 | 0.000 |
| Residents under poverty level | 0.003 | 0.000 | 0.000 | 0.003 | 0.004 |
| Female | 0.684 | 0.003 | 0.000 | 0.678 | 0.690 |
| Black, non-Hispanic race vs. white, non-Hispanic | -1.005 | 0.006 | 0.000 | -1.017 | -0.994 |
| Hispanic ethnicity vs. white, non-Hispanic | -0.866 | 0.008 | 0.000 | -0.881 | -0.852 |
| Other, non-Hispanic vs. white, non-Hispanic | -0.798 | 0.008 | 0.000 | -0.814 | -0.782 |
| Disabled (original reason for Medicare eligibility) | 0.786 | 0.006 | 0.000 | 0.775 | 0.798 |
| Dual eligible for Medicaid | 0.176 | 0.005 | 0.000 | 0.167 | 0.185 |
| Small town vs. isolated rural | 0.017 | 0.009 | 0.075 | -0.002 | 0.035 |
| Micropolitan vs. isolated rural | 0.227 | 0.009 | 0.000 | 0.210 | 0.244 |
| Metropolitan vs. isolated rural | 0.484 | 0.008 | 0.000 | 0.468 | 0.499 |
| Hospital referral region (suppressed) |  |  |  |  |  |
| Constant | 0.249 | 0.026 | 0.000 | 0.199 | 0.299 |
| **P-value for difference between nurse practitioner vs. physician** | F=377,562.1 | P<0.000 |  |  |  |

**Table 17: Number of visits to a nurse practitioner, 2017 (Figure 3)**

| R-squared | 0.333 |  |  |  |  |
| --- | --- | --- | --- | --- | --- |
| Number of observations | 6,164,090 |  |  |  |  |
|  | **Coef** | **SE** | **P-value** | **LL CI** | **LL CI** |
| Nurse practitioner vs. physician | 1.797 | 0.006 | 0.000 | 1.786 | 1.808 |
| 1 to 2 vs 0 hierarchical condition categories | 0.350 | 0.004 | 0.000 | 0.342 | 0.359 |
| 3 to 5 vs 0 hierarchical condition categories | 0.878 | 0.005 | 0.000 | 0.868 | 0.888 |
| 6+ vs 0 hierarchical condition categories | 1.598 | 0.007 | 0.000 | 1.585 | 1.611 |
| Nurse practitioner vs. physician * hierarchical condition categories |  |  |  |  |  |
| Nurse practitioner*1-2 conditions | 2.345 | 0.007 | 0.000 | 2.330 | 2.360 |
| Nurse practitioner*3-5 conditions | 4.726 | 0.009 | 0.000 | 4.708 | 4.744 |
| Nurse practitioner*6+ conditions | 6.528 | 0.012 | 0.000 | 6.505 | 6.551 |
| Mean age, years | 0.025 | 0.000 | 0.000 | 0.025 | 0.026 |
| Over 85 | 0.068 | 0.006 | 0.000 | 0.056 | 0.079 |
| Under 65 | 0.056 | 0.007 | 0.000 | 0.041 | 0.070 |
| Median household income | 0.000 | 0.000 | 0.000 | 0.000 | 0.000 |
| Residents under poverty level | 0.002 | 0.000 | 0.000 | 0.002 | 0.002 |
| Female | 0.295 | 0.003 | 0.000 | 0.290 | 0.301 |
| Black, non-Hispanic race vs. white, non-Hispanic | -0.216 | 0.006 | 0.000 | -0.227 | -0.205 |
| Hispanic ethnicity vs. white, non-Hispanic | -0.262 | 0.008 | 0.000 | -0.278 | -0.246 |
| Other, non-Hispanic vs. white, non-Hispanic | -0.228 | 0.010 | 0.000 | -0.247 | -0.208 |
| Disabled (original reason for Medicare eligibility) | 0.300 | 0.005 | 0.000 | 0.290 | 0.310 |
| Dual eligible for Medicaid | 1.216 | 0.004 | 0.000 | 1.208 | 1.224 |
| Small town vs. isolated rural | -0.054 | 0.008 | 0.000 | -0.070 | -0.038 |
| Micropolitan vs. isolated rural | -0.008 | 0.007 | 0.294 | -0.022 | 0.007 |
| Metropolitan vs. isolated rural | 0.040 | 0.007 | 0.000 | 0.026 | 0.053 |
| Hospital referral region (suppressed) |  |  |  |  |  |
| Constant | -0.824 | 0.024 | 0.000 | -0.870 | -0.778 |
| **P-value for difference between nurse practitioner vs. physician** | F=1,770,000 | P<0.000 |  |  |  |

**Table 18: Number of visits to a specialist, 2017 (Figure 3)**

| R-squared | 0.170 |  |  |  |  |
| --- | --- | --- | --- | --- | --- |
| Number of observations | 16,573,459 |  |  |  |  |
|  | **Coef** | **SE** | **P-value** | **LL CI** | **LL CI** |
| Nurse practitioner vs. physician | -0.681 | 0.010 | 0.000 | -0.701 | -0.661 |
| 1 to 2 vs 0 hierarchical condition categories | 2.590 | 0.003 | 0.000 | 2.583 | 2.596 |
| 3 to 5 vs 0 hierarchical condition categories | 5.642 | 0.004 | 0.000 | 5.634 | 5.651 |
| 6+ vs 0 hierarchical condition categories | 7.269 | 0.006 | 0.000 | 7.257 | 7.282 |
| Nurse practitioner vs. physician * hierarchical condition categories |  |  |  |  |  |
| Nurse practitioner*1-2 conditions | -1.096 | 0.013 | 0.000 | -1.122 | -1.070 |
| Nurse practitioner*3-5 conditions | -2.433 | 0.016 | 0.000 | -2.464 | -2.402 |
| Nurse practitioner*6+ conditions | -3.036 | 0.019 | 0.000 | -3.073 | -2.998 |
| Mean age, years | 0.006 | 0.000 | 0.000 | 0.005 | 0.006 |
| Over 85 | -1.122 | 0.006 | 0.000 | -1.134 | -1.111 |
| Under 65 | 0.236 | 0.007 | 0.000 | 0.221 | 0.250 |
| Median household income | 0.000 | 0.000 | 0.000 | 0.000 | 0.000 |
| Residents under poverty level | 0.002 | 0.000 | 0.000 | 0.002 | 0.002 |
| Female | 0.260 | 0.003 | 0.000 | 0.255 | 0.265 |
| Black, non-Hispanic race vs. white, non-Hispanic | -0.713 | 0.005 | 0.000 | -0.723 | -0.703 |
| Hispanic ethnicity vs. white, non-Hispanic | -0.764 | 0.007 | 0.000 | -0.777 | -0.750 |
| Other, non-Hispanic vs. white, non-Hispanic | -0.848 | 0.008 | 0.000 | -0.863 | -0.833 |
| Disabled (original reason for Medicare eligibility) | 0.524 | 0.005 | 0.000 | 0.514 | 0.534 |
| Dual eligible for Medicaid | -0.733 | 0.004 | 0.000 | -0.741 | -0.724 |
| Small town vs. isolated rural | -0.064 | 0.009 | 0.000 | -0.081 | -0.047 |
| Micropolitan vs. isolated rural | 0.093 | 0.008 | 0.000 | 0.077 | 0.108 |
| Metropolitan vs. isolated rural | 0.406 | 0.007 | 0.000 | 0.391 | 0.420 |
| Hospital referral region (suppressed) |  |  |  |  |  |
| Constant | 1.950 | 0.023 | 0.000 | 1.905 | 1.996 |
| **P-value for difference between nurse practitioner vs. physician** | F=99,131.030 | P<0.000 |  |  |  |

**Table 19: Number of providers seen, 2017 (Figure 3)**

| R-squared | 0.195 |  |  |  |  |
| --- | --- | --- | --- | --- | --- |
| Number of observations | 21,039,399 |  |  |  |  |
|  | **Coef** | **SE** | **P-value** | **LL CI** | **LL CI** |
| Nurse practitioner vs. physician | -0.008 | 0.003 | 0.012 | -0.015 | -0.002 |
| 1 to 2 vs 0 hierarchical condition categories | 1.390 | 0.001 | 0.000 | 1.388 | 1.393 |
| 3 to 5 vs 0 hierarchical condition categories | 2.942 | 0.002 | 0.000 | 2.938 | 2.946 |
| 6+ vs 0 hierarchical condition categories | 3.880 | 0.003 | 0.000 | 3.874 | 3.885 |
| Nurse practitioner vs. physician * hierarchical condition categories |  |  |  |  |  |
| Nurse practitioner*1-2 conditions | 0.113 | 0.005 | 0.000 | 0.104 | 0.122 |
| Nurse practitioner*3-5 conditions | 0.032 | 0.006 | 0.000 | 0.020 | 0.043 |
| Nurse practitioner*6+ conditions | 0.316 | 0.008 | 0.000 | 0.301 | 0.331 |
| Mean age, years | 0.009 | 0.000 | 0.000 | 0.009 | 0.009 |
| Over 85 | -0.730 | 0.002 | 0.000 | -0.735 | -0.725 |
| Under 65 | -0.102 | 0.003 | 0.000 | -0.108 | -0.096 |
| Median household income | 0.000 | 0.000 | 0.000 | 0.000 | 0.000 |
| Residents under poverty level | 0.000 | 0.000 | 0.005 | 0.000 | 0.000 |
| Female | 0.324 | 0.001 | 0.000 | 0.322 | 0.326 |
| Black, non-Hispanic race vs. white, non-Hispanic | -0.569 | 0.002 | 0.000 | -0.573 | -0.565 |
| Hispanic ethnicity vs. white, non-Hispanic | -0.514 | 0.003 | 0.000 | -0.520 | -0.508 |
| Other, non-Hispanic vs. white, non-Hispanic | -0.625 | 0.003 | 0.000 | -0.631 | -0.619 |
| Disabled (original reason for Medicare eligibility) | 0.138 | 0.002 | 0.000 | 0.133 | 0.142 |
| Dual eligible for Medicaid | -0.518 | 0.002 | 0.000 | -0.521 | -0.514 |
| Small town vs. isolated rural | 0.021 | 0.004 | 0.000 | 0.014 | 0.028 |
| Micropolitan vs. isolated rural | 0.185 | 0.003 | 0.000 | 0.178 | 0.191 |
| Metropolitan vs. isolated rural | 0.469 | 0.003 | 0.000 | 0.463 | 0.475 |
| Hospital referral region (suppressed) |  |  |  |  |  |
| Constant | 1.550 | 0.010 | 0.000 | 1.531 | 1.569 |
| **P-value for difference between nurse practitioner vs. physician** | F=838.32 | P=0.000 |  |  |  |

**Table 20: Percentage of beneficiaries who get the plurality of their care from a nurse practitioner by state, 2017**


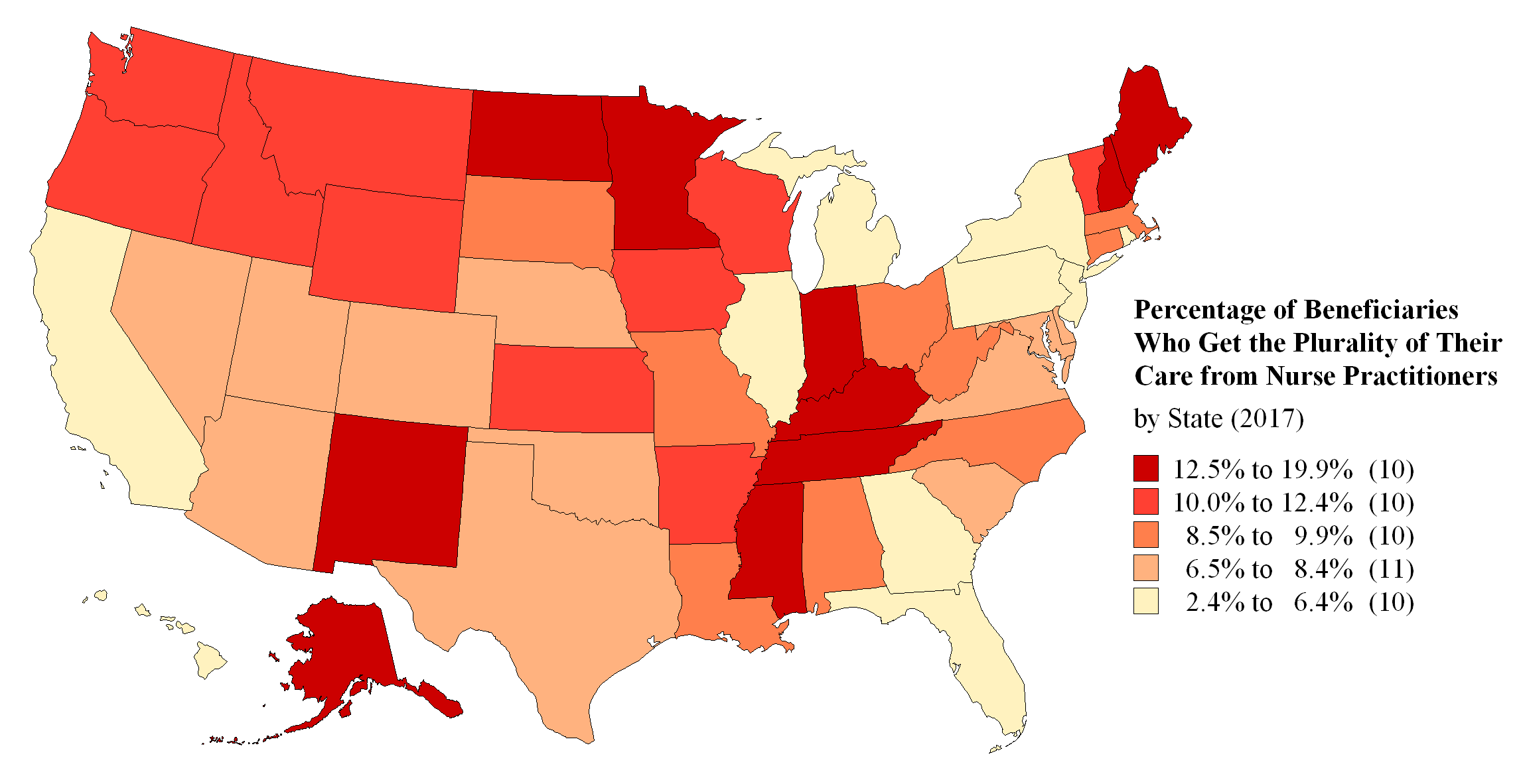


| **State** | **% of beneficiaries assigned to NP** | **State** | **% of beneficiaries assigned to NP** | **State** | **% of beneficiaries assigned to NP** |
| --- | --- | --- | --- | --- | --- |
| Alabama | 8.5% | Kentucky | 17.8% | North Dakota | 18.2% |
| Alaska | 13.9% | Louisiana | 9.5% | Ohio | 9.5% |
| Arizona | 8.3% | Maine | 16.1% | Oklahoma | 8.3% |
| Arkansas | 10.4% | Maryland | 6.8% | Oregon | 11.5% |
| California | 2.4% | Massachusetts | 8.9% | Pennsylvania | 5.3% |
| Colorado | 7.7% | Michigan | 6.0% | Rhode Island | 5.7% |
| Connecticut | 9.1% | Minnesota | 13.0% | South Carolina | 7.3% |
| Delaware | 6.5% | Mississippi | 19.9% | South Dakota | 9.4% |
| Dist. Columbia | 8.8% | Missouri | 9.7% | Tennessee | 15.7% |
| Florida | 5.7% | Montana | 12.1% | Texas | 6.7% |
| Georgia | 6.0% | Nebraska | 8.4% | Utah | 7.1% |
| Hawaii | 3.2% | Nevada | 7.6% | Vermont | 11.7% |
| Idaho | 11.2% | New Hampshire | 13.7% | Virginia | 7.5% |
| Illinois | 6.4% | New Jersey | 4.4% | Washington | 10.4% |
| Indiana | 12.7% | New Mexico | 14.3% | West Virginia | 9.2% |
| Iowa | 12.0% | New York | 5.7% | Wisconsin | 10.0% |
| Kansas | 10.8% | North Carolina | 9.4% | Wyoming | 10.9% |

**Table 21: Correlation matrix**

|  | 1 | 2 | 3 | 4 | 5 | 6 | 7 | 8 | 9 | 10 | 11 | 12 | 13 | 14 | 15 | 16 | 17 | 18 | 19 |
| --- | --- | --- | --- | --- | --- | --- | --- | --- | --- | --- | --- | --- | --- | --- | --- | --- | --- | --- | --- |
| Nurse practitioner vs. physician | 1.00 |  |  |  |  |  |  |  |  |  |  |  |  |  |  |  |  |  |  |
| Mean age | -0.04 | 1.00 |  |  |  |  |  |  |  |  |  |  |  |  |  |  |  |  |  |
| Median household income | -0.07 | 0.08 | 1.00 |  |  |  |  |  |  |  |  |  |  |  |  |  |  |  |  |
| Residents under poverty level | 0.05 | -0.08 | -0.65 | 1.00 |  |  |  |  |  |  |  |  |  |  |  |  |  |  |  |
| Number HCCs | 0.05 | 0.07 | -0.05 | 0.05 | 1.00 |  |  |  |  |  |  |  |  |  |  |  |  |  |  |
| Over 85 | 0.03 | 0.53 | 0.01 | -0.01 | 0.10 | 1.00 |  |  |  |  |  |  |  |  |  |  |  |  |  |
| Under 65 | 0.07 | -0.72 | -0.11 | 0.11 | 0.06 | -0.14 | 1.00 |  |  |  |  |  |  |  |  |  |  |  |  |
| Female | 0.03 | 0.08 | 0.00 | 0.00 | -0.05 | 0.07 | -0.05 | 1.00 |  |  |  |  |  |  |  |  |  |  |  |
| Race | -0.02 | -0.08 | -0.01 | 0.11 | 0.03 | -0.03 | 0.08 | 0.01 | 1.00 |  |  |  |  |  |  |  |  |  |  |
| Disabled | 0.07 | -0.60 | -0.14 | 0.13 | 0.14 | -0.16 | 0.76 | -0.06 | 0.09 | 1.00 |  |  |  |  |  |  |  |  |  |
| Dual eligible for Medicaid | 0.10 | -0.31 | -0.14 | 0.17 | 0.17 | 0.00 | 0.42 | 0.04 | 0.24 | 0.41 | 1.00 |  |  |  |  |  |  |  |  |
| Died in the year | 0.06 | 0.14 | -0.02 | 0.01 | 0.34 | 0.17 | -0.03 | -0.01 | -0.01 | -0.01 | 0.06 | 1.00 |  |  |  |  |  |  |  |
| Urbanicity | -0.08 | 0.02 | 0.28 | -0.17 | 0.02 | 0.01 | -0.03 | 0.02 | 0.09 | -0.04 | -0.03 | -0.01 | 1.00 |  |  |  |  |  |  |
| Coronary artery disease | 0.00 | 0.06 | -0.02 | 0.01 | 0.35 | 0.03 | -0.02 | -0.05 | 0.00 | 0.01 | 0.02 | 0.10 | 0.00 | 1.00 |  |  |  |  |  |
| Congestive heart failure | 0.03 | 0.14 | -0.03 | 0.03 | 0.56 | 0.14 | -0.03 | -0.03 | 0.00 | 0.02 | 0.06 | 0.20 | 0.00 | 0.25 | 1.00 |  |  |  |  |
| Diabetes | 0.01 | 0.02 | -0.06 | 0.05 | 0.39 | -0.03 | 0.00 | -0.05 | 0.10 | 0.06 | 0.08 | 0.04 | -0.01 | 0.10 | 0.14 | 1.00 |  |  |  |
| Cancer | -0.03 | 0.07 | 0.02 | -0.02 | 0.25 | 0.00 | -0.06 | -0.06 | -0.02 | -0.04 | -0.04 | 0.12 | 0.02 | 0.02 | 0.04 | 0.02 | 1.00 |  |  |
| Chronic obstructive pulmonary disease | 0.03 | 0.04 | -0.06 | 0.04 | 0.42 | 0.02 | 0.02 | -0.02 | -0.03 | 0.08 | 0.10 | 0.12 | -0.03 | 0.12 | 0.24 | 0.07 | 0.06 | 1.00 |  |
| End stage renal disease | 0.00 | -0.09 | -0.03 | 0.04 | 0.25 | -0.02 | 0.11 | -0.03 | 0.08 | 0.12 | 0.08 | 0.06 | 0.01 | 0.06 | 0.12 | 0.10 | 0.00 | 0.03 | 1.00 |

**References**

1. Medicare program; Medicare shared savings program; accountable care organizations-pathways to success and extreme and uncontrollable circumstances policies for performance year 2017, 83 FR §67816 (2018).

2. Centers for Medicare and Medicaid Services. Medicare shared savings program: Shared savings and losses and assignment methodology. <https://www.cms.gov/Medicare/Medicare-Fee-for-Service-Payment/sharedsavingsprogram/Downloads/Shared-Savings-Losses-Assignment-Spec-V5.pdf>. Published 2017. Updated April 2017. Accessed December 24, 2017.
